# Interplay between Schizophrenia Polygenic Risk Score and Childhood Adversity in First-Presentation Psychotic Disorder: A Pilot Study

**DOI:** 10.1371/journal.pone.0163319

**Published:** 2016-09-20

**Authors:** Antonella Trotta, Conrad Iyegbe, Marta Di Forti, Pak C. Sham, Desmond D. Campbell, Stacey S. Cherny, Valeria Mondelli, Katherine J. Aitchison, Robin M. Murray, Evangelos Vassos, Helen L. Fisher

**Affiliations:** 1 Department of Psychosis Studies, Institute of Psychiatry, Psychology & Neuroscience, King’s College London, London, United Kingdom; 2 MRC Social, Genetic and Developmental Psychiatry Centre, Institute of Psychiatry, Psychology & Neuroscience, King’s College London, London, United Kingdom; 3 Department of Psychiatry, University of Hong Kong, Hong Kong, Special Administrative Region of China; 4 Centre for Genomic Sciences, University of Hong Kong, Hong Kong, Special Administrative Region of China; 5 Department of Psychological Medicine, Institute of Psychiatry, Psychology & Neuroscience, King’s College London, London, United Kingdom; 6 Departments of Psychiatry and Medical Genetics, University of Alberta, Edmonton, Alberta, Canada; University of Texas Health Science Center at Houston, UNITED STATES

## Abstract

A history of childhood adversity is associated with psychotic disorder, with an increase in risk according to number or severity of exposures. However, it is not known why only some exposed individuals go on to develop psychosis. One possibility is pre-existing genetic vulnerability. Research on gene-environment interaction in psychosis has primarily focused on candidate genes, although the genetic effects are now known to be polygenic. This pilot study investigated whether the effect of childhood adversity on psychosis is moderated by the polygenic risk score for schizophrenia (PRS). Data were utilised from the Genes and Psychosis (GAP) study set in South London, UK. The GAP sample comprises 285 first-presentation psychosis cases and 256 unaffected controls with information on childhood adversity. We studied only white subjects (80 cases and 110 controls) with PRS data, as the PRS has limited predictive ability in patients of African ancestry. The occurrence of childhood adversity was assessed with the Childhood Experience of Care and Abuse Questionnaire (CECA.Q) and the PRS was based on genome-wide meta-analysis results for schizophrenia from the Psychiatric Genomics Consortium. Higher schizophrenia PRS and childhood adversities each predicted psychosis status. Nevertheless, no evidence was found for interaction as departure from additivity, indicating that the effect of polygenic risk scores on psychosis was not increased in the presence of a history of childhood adversity. These findings are compatible with a multifactorial threshold model in which both genetic liability and exposure to environmental risk contribute independently to the etiology of psychosis.

## Introduction

One widely replicated environmental risk factor for psychosis is exposure to adverse experiences in childhood [1,2], such as physical or sexual abuse, or parental separation. Furthermore, the literature suggests that adversities are damaging if they are overwhelming and persistent, as demonstrated by a high rate of multiple childhood traumatic experiences in people with psychosis [3]. It is not known why only a small proportion of individuals who experience adversity in childhood later develop psychosis. One possibility is pre-existing genetic vulnerability. In the absence of direct genetic data, researchers have used familial aggregation of psychiatric disorders as proxy measures of genetic risk. However, most of the studies involving familial liability have been restricted to general population samples and results are still controversial [4–9]. Only two studies have investigated the interplay between childhood adversity and familial risk for mental health problems in a first-episode psychosis sample [10,11] and found no evidence of gene-environment interaction. To date, interactions between potential molecular genetic susceptibility and exposure to childhood adversity in predicting development of psychosis have mainly focused on candidate genes such as *FKBP5* [12,13], *BDNF* [14,15], and *COMT* [16].

Recent research indicates that many (probably thousands) of genetic loci confer risk for psychosis and that common variation in the form of SNPs can be used to tag these loci [17]. Although individually small, in aggregate these effects are quite predictive of risk, with one method of aggregation being polygenic risk scores [18]. The capacity of a polygenic risk score (PRS) to predict onset of schizophrenia has been established and has been found to explain up to 7% of additive genetic liability for this severe mental illness [17]. However, very little has been done to examine the interaction between PRS and childhood adversity in predicting psychiatric disorders. Those studies that do exist have explored this interaction in relation to risk of depression [19,20], health problems and alcohol use [21,22], and smoking behaviour [23], and the findings consistently support the presence of a GxE interaction. However, no studies to date have explored whether a PRS modifies the association between childhood adversity and onset of psychotic disorders.

Therefore, the current pilot study aimed to investigate associations between polygenic risk scores, childhood adversity, and psychosis case status in a sample of first-presentation psychosis cases and unaffected community controls. We have previously shown in this sample that different forms of childhood adversity are associated with presence of psychotic disorder [11], and that a schizophrenia PRS accounts for 9% of the liability to psychotic disorder [24]. We hypothesized that cumulative exposure to adversity during childhood would moderate the association between a schizophrenia PRS and psychotic disorder.

## Methods

### Study Design and Participants

The sample was drawn from cases and controls who participated in the Genetics and Psychosis (GAP) study from the Lambeth, Southwark, Lewisham and Croydon adult in-patient and out-patient units of the South London & Maudsley (SLAM) Mental Health National Health Service (NHS) Foundation Trust. Participants from this study were included if they had been assessed regarding childhood adversity and provided an analysable sample of DNA. Inclusion criteria for cases were: aged 18–65 years, presenting to psychiatric services for the first time with a psychotic disorder (codes F20–29 and F30–33 from the International Classification of Diseases [ICD-10]) [25], and resident within tightly defined catchment areas in Southeast London, UK. Exclusion criteria were: organic psychosis; intelligence quotient (IQ) under 70; previous contact with services for psychosis, and transient psychotic symptoms resulting from acute drug intoxication. ICD-10 diagnoses were determined using data from the Schedules for Clinical Assessment in Neuropsychiatry (SCAN) [26]. Validation of clinical diagnosis was obtained using the computerized Operational Criteria system (version 2004) [27]. All diagnoses were performed by qualified psychologists and psychiatrists, subject to comprehensive training and achievement of good inter-rater reliability (κ = 0.91). Patients diagnosed as having bipolar disorder or major depression with psychotic symptoms were included in the affective psychosis group (ICD-10 codes F30-33), while patients with schizophrenia, schizophreniform disorder, and schizoaffective disorder formed the schizophrenia-spectrum disorders group (ICD-10 codes F20-29).

Controls were aged 18–65 years and recruited from the local population living in the area served by the Trust, by means of internet and newspaper advertisements, and distribution of leaflets at train stations, shops, and job centres. Efforts were made to obtain a control sample that was representative of the general population in age, gender, ethnicity, educational qualifications, and employment status. The Psychosis Screening Questionnaire (PSQ) [28] was administered to all potential control group participants; individuals were excluded if they met criteria for a psychotic disorder.

### Measures

#### Childhood adversity

The Childhood Experience of Care and Abuse Questionnaire (CECA.Q) [29] was employed to retrospectively elicit information on a range of adverse childhood experiences from participants. Physical abuse by the main mother and father figures (usually, but not necessarily, the biological parents), sexual abuse by any adult or an individual at least 5 years older than the recipient, separation from a parent for at least 6 months, death of a parent, taken into institutional care, and number of family arrangements, all prior to 17 years of age, were assessed. The CECA.Q was read out to all participants during face-to-face interviews to improve the accuracy of the fixed category responses obtained. This questionnaire has been shown to have good internal consistency [30], satisfactory levels of test-retest reliability over 7 years in a similar psychosis sample [31], and reasonable concurrent validity with existing measures [29–31].

#### Genotyping and quality control

DNA was obtained from all participants that completed the CECA.Q. Seventy-five percent of DNA samples used originated from blood and 25% from cheek swabs. All the genotypes underwent extensive manual quality control (QC) using GenomeStudio. Only swabs when DNA met quality standards were included in the analysis.

During QC, SNPs were excluded that: deviated from Hardy–Weinberg equilibrium with a P-value <1×10^−5^ in controls; had a minor allele frequency <1%; or were missing in >1% of individuals. Individuals were excluded that: had discordant gender information; a genotyping failure in more than 1% of SNPs; or if there was genetic evidence of relatedness with other individuals included in the sample. Principal component (PC) analysis was applied via EIGENSTRAT [32] to model population structure and any outlier individuals were excluded. Ten PCs were included as covariates in the genetic analyses to control for the effects of population stratification.

#### Polygenic risk score calculation

The polygenic risk scores were constructed using the results from a large mega-analysis from the Schizophrenia Working Group of the Psychiatric Genomics Consortium [17]. For the purpose of the analysis, PRS was constructed based on the PGC2 leave-one-out discovery dataset, excluding the WTCCC2 sample (http://www.wtccc.org.uk/ccc2/) as this contained GAP participants. The GAP cohort therefore represents an independent validation dataset. A subset of around 9.5 million imputed autosomal SNPs was selected. SNPs were pruned using the ‘clumping’ procedure implemented in PLINK [33], which retains from each LD block those SNPs most associated with schizophrenia in the discovery set and removes SNPs in high LD showing less evidence of association (maximum r^2^ = 0.1, window = 250kb, filtering for significance). Using ten significance thresholds, the number of risk alleles possessed by each individual in the target sample was calculated, weighted by the log odds ratio from the discovery sample, and aggregated into a polygenic score [17].

Following QC, there were 80 white European first-presentation psychosis cases and 110 white European unaffected controls with both genome-wide genotype data and childhood adversity information available. We could only include participants of white European parentage to ensure a meaningful analysis of interaction as the schizophrenia PRS in GAP participants with African ancestry was much less predictive of psychosis (1% of variance explained) [24].

### Ethics

This study was part of the GAP study, which was granted ethical approval by the South London and Maudsley and Institute of Psychiatry Local Research Ethics Committee. All cases and control subjects included in the study gave informed written consent, after reading a detailed information sheet, to participate in the study and to publication of data originating from the study.

### Statistical analysis

A composite variable was computed to summarise how many of the different adversities had been experienced by each individual, following the guidelines published by Bifulco et al. [29]. This ‘total adversity’ score involved summing the dichotomous CECA.Q severity subscale scores (range 0–6) and then recoding the total into an ordinal scale of 0 (none), 1 (single adverse experience), and 2 (multiple adverse experiences).

The association between the schizophrenia PRS and the presence or absence of (i) psychotic disorder, and (ii) childhood adversity (i.e., gene–environment correlation) was tested using a logistic regression model, controlling for population stratification, sex, age and education level, because such factors could potentially bias the results. This analysis was performed separately for cases and controls in order to test if the PRS was associated with childhood adversity in both groups. The cumulative effect of childhood adversity was also tested for association with case/control status.

Possible interaction between childhood adversity and PRS was investigated using an additive model to test interaction as departure from additivity. This means that the combined effect of PRS and environment differs from the sum of their individual effects. Departure from additivity seems to be more in line with biological interaction [34]. It is also more relevant to clinical and public health implications. Interaction as departure from additivity was tested using linear regression of psychosis case/control status on the interaction term, with covariates of age, gender, level of education and 10 PCs to take population stratification into account. The interaction model was also adjusted for PC × environment and PC × PRS interactions [35]. Effects were considered significant when *p*-values were <0.05 or when 95% confidence intervals did not contain zero. All analyses were conducted using R (http://www.r-project.org).

## Results

### Sample characteristics

Sample characteristics are shown in Table 1. Information on childhood adversities was available for 285 first-presentation psychosis patients and 256 unaffected controls. Compared with controls, and in line with what would be expected, psychosis cases had a lower level of education (*p*<0.001), and were more often of non-white ethnicity (*p*<0.001). There was no significant difference between psychosis cases and controls in terms of gender (*p* = 0.065) and age (*p* = 0.733).

**Table 1:** Genes and Psychosis (GAP) study sample characteristics.

	Overall sample					PRS subsample				
Demographic variable	Cases	Controls				Cases	Controls			
	(*N* = 285)	(*N* = 256)	χ^2^	df		(*N* = 80)	(*N* = 110)			
	*n* (%)	*n* (%)	χ^2^	df	*p*	*n* (%)	*n* (%)	χ^2^	df	*p*
**Gender**			2.57	1	0.065			0.10	1	0.756
Men	172 (60.4)	137 (53.5)				44 (55.0)	58 (52.7)			
Women	113 (39.6)	119 (46.5)				36 (45.0)	52 (47.3)			
**Ethnicity**^a^			**32.60**	**5**	**<0.001**					
White British	72 (25.3)	102 (39.8)								
Black Caribbean	56 (19.6)	39 (15.2)								
Black African	65 (22.8)	32 (12.5)								
White Other	30 (10.5)	50 (19.5)								
Asian (all)	24 (8.4)	16 (6.3)								
Other	38 (13.3)	17 (6.6)								
**Level of education**			**76.73**	**4**	**<0.001**			**26.56**	4	**<0.001**
No qualifications	48 (17.6)	7 (3.0)				19 (23.7)	2 (2.0)			
GCSE/O level	64 (23.5)	23 (10.0)				12 (15.0)	12 (12.0)			
A level	40 (14.7)	53 (22.9)				11 (13.7)	22 (22.0)			
Vocational	66 (24.3)	37 (16.0)				16 (20.0)	14 (14.0)			
University or professional qualifications	54 (19.9)	111 (48.1)				22 (27.5)	50 (50.0)			
**Age in years**			t = 0.342	536	0.733			t = 0.79	187	0.431
Mean (S.D.)	28.9 (9.3)	29.2 (9.9)				28.8 (9.5)	30 (10.4)			

**Notes:** df, degrees of freedom; GCSE, General Certificate of Secondary Education; PRS, polygenic risk score; S.D., standard deviation. Figures in bold indicate *p*<0.05.

^a^This comparison is not applicable for the PRS subsample as both cases and controls were selected to be of White European ancestry.

In the subset of the GAP sample with PRS data (80 white first-episode psychosis cases and 110 white controls), there were no significant differences between the psychosis cases and community controls in terms of gender and age, but controls were more likely to hold university or professional qualifications than cases. This underlines the importance of controlling for educational level in the subsequent analyses. This subsample did not differ in terms of gender (cases: χ^2^ = 1.33, *p* = 0.249; controls: χ^2^ = 0.03, *p* = 0.900) or age (cases: *t* = 0.09, *p* = 0.930; controls: *t* = -1.14, *p* = 0.256) from those with no PRS data available.

Baseline diagnoses were available on 218 psychosis cases with a complete CECA.Q from the GAP study. Of these cases, 150 (68.8%) had an ICD-10 diagnosis of schizophrenia-spectrum disorders, 42 (19.3%) of affective psychosis, and the rest of the cases (n = 26, 11.9%) were classified as ‘other psychosis’. Similarly, in the subsample with PRS data available, 37 (60.7%) cases had an ICD-10 diagnosis of schizophrenia-spectrum disorders, 15 (24.5%) of affective psychosis, and 9 cases (14.8%) were classified as ‘other psychosis’.

### Childhood adversity, polygenic score, and risk for psychotic disorder

Psychosis cases in the full CECA.Q sample had experienced significantly more childhood adversities than controls (Table 2). The most prevalent forms of adversity prior to the age of 17 years amongst both psychosis cases (56.6%) and unaffected controls (35.7%) were separation from biological father or mother for at least six months. First-presentation psychosis patients were more than two times more likely to report exposure to two or more childhood adversities compared with controls (*p* = 0.003). In fact, a score test for trend provided evidence for a linear trend (*z* = 4.97, *p*<0.001), indicating a dose-response effect for repeated adverse experiences.

**Table 2:** Prevalence of childhood adversities amongst first-presentation psychosis cases and unaffected controls.

Total adversity exposure	Cases	Controls	Unadjusted OR	95% CI	*p*	Adjusted OR*	95% CI	*p*
	*n/N* (%)	*n/N* (%)						
**Overall sample**								
None	82/285 (28.8)	130/256 (50.8)	1.0	-	-	1.0	-	-
One type	121/285 (42.4)	81/256 (31.6)	**2.37**	1.60–3.51	**<0.001**	**2.01**	1.30–3.11	**0.002**
Two or more types	82/285 (28.8)	45/256 (17.6)	**2.88**	1.83–4.56	**<0.001**	**2.17**	1.31–3.61	**0.003**
**PRS sample**								
None	34/86 (39.6)	66/110 (59.1)	1.0	-	-	1.0	-	-
One type	31/86 (36.0)	28/110 (25.4)	**2.15**	1.11–4.15	**0.023**	1.72	0.85–3.50	0.133
Two or more types	21/86 (24.4)	16/110 (15.5)	**2.55**	1.18–5.51	**0.017**	2.06	0.89–4.74	0.090

**Notes:** CI, confidence interval. OR, odds ratio. PRS, polygenic risk score. Figures in bold indicate *p*<0.05.

*Adjusted for gender, age at interview, ethnicity and level of education.

The cumulative effect of childhood adversity on psychosis status also held in the subsample with PRS data (*z* = 2.58, *p* = 0.010). The association with psychosis was slightly stronger for participants who reported multiple (OR = 2.55) than single (OR = 2.15) adverse childhood experiences in this subsample. However, after adjusting for demographic confounders, the association between single or multiple childhood adversities and psychosis remained only at a trend level of significance.

Furthermore, higher polygenic scores significantly predicted psychosis case status in this subsample (adjusted *b* = 7.68, 95% CI 3.69–11.66, *p*<0.001), and the association held when the sample was restricted to those cases with an ICD-10 diagnosis of schizophrenia-spectrum disorders (adjusted *b* = 8.86, 95% CI 3.55–14.16, *p* = 0.001).

#### Gene-environment interaction

To rule out the possibility of gene-environment correlation, we examined the associations between PRS and childhood adversity measures, adjusting for PCs and demographic confounders. Table 3 shows the results of gene-environment correlations in cases and controls, respectively (results without adjustment for education are provided in S1 Table). In both first-presentation psychosis cases and unaffected controls, higher polygenic scores were not significantly associated with childhood adversity. Therefore, no evidence of gene-environment correlation was found in either group. Sensitivity analyses confirmed no association between PRS and childhood adversity in those cases with a diagnosis of schizophrenia-spectrum disorders (results are provided in S2 Table).

**Table 3:** Associations between the schizophrenia polygenic risk score and reports of childhood adversity.

Gene–Environment correlation	Adjusted b*	95% CI	*p*	Adjusted b**	95% CI	*p*
Psychosis Cases	2.26	-3.87–8.38	0.470	1.71	-5.37–8.78	0.636
Unaffected Controls	0.95	-3.35–5.26	0.664	2.77	-1.92–7.47	0.247

**Notes:** CI, confidence interval. *b*, logistic regression coefficient.

*adjusted for ten principal components.

**further adjusted for gender, age at interview and education level.

The results of interaction between PRS and multiple childhood adversities on the presence of psychotic disorder showed no evidence for interaction as departure from additivity (Table 4; and without adjustment for education in S3 Table), indicating that the cumulative effect of the number of childhood adversities reported on first presentation for psychosis was not moderated by the schizophrenia PRS (*p* = 0.918). Sensitivity analyses also confirmed no interaction between PRS and childhood adversity in those cases with a diagnosis of schizophrenia-spectrum disorders (results are provided in S4 Table).

**Table 4:** Interaction between the schizophrenia polygenic risk score and reports of childhood adversity on presence of psychotic disorder.

Gene–Environment Interaction	b*	Std. Error	*p*	Adjusted b**	Std. Error	*p*
PRS	**0.39**	0.14	**0.004**	**0.43**	0.14	**0.002**
Childhood adversity	0.21	0.42	0.623	-0.05	0.43	0.902
PRS* Childhood adversity	0.04	0.40	0.918	-0.20	0.41	0.632

**Notes:** PRS, Childhood adversity and their interaction were standardised by subtracting the mean and dividing by the standard deviation prior to fitting the model.

*b*, linear regression coefficient. PRS, polygenic risk score. Std. Error, Standard Error. Figures in bold indicate *p*<0.05.

*adjusted for ten principal components.

**further adjusted for gender, age at interview and education level.

## Discussion

There has been a shift in psychiatric genetics towards using polygenic risk scores rather than candidate genes to index genetic risk for mental illness. In this study, polygenic risk scores derived from a schizophrenia GWAS by the Psychiatric Genomics Consortium [17] were tested for their ability to predict psychosis case/control status in this ethnically-restricted sample for whom data on early life exposures were available. Moreover, childhood adversity was tested for an interaction with this PRS. A direct molecular measure of genetic risk was used to show that the association between childhood adversity and psychosis is unlikely to be explained by gene-environment correlation. We defined this as a pilot study as these exploratory analyses were conducted as a “small-scale test of the methods and procedures to be used on a larger scale” [36]. The fundamental purpose of conducting this pilot study was, in fact, to examine the feasibility of a using a polygenic GxE interaction approach in psychosis in order to conduct analyses in a future larger-scale study.

As expected, the schizophrenia PRS predicted psychotic disorder in this GAP subsample, consistent with results in the full sample [24]. Similarly, in the GAP European subsample, cases had on average higher adjusted PRS than controls, with standardized mean difference of 0.54, following correction for population stratification, and the association significantly held for both schizophrenia (R^2^ = 16.3%, *p* = 3.7*10^−7^) and other psychoses groups (R^2^ = 2.7%, *p* = 0.03) [24]. This is in line with previous studies [23] demonstrating that cumulative genetic risk predicted case–control status for psychosis across independent samples at a high significance level. The predictive power of the PRS, based on the much larger PGC dataset, allowed these questions to be investigated in a smaller dataset than would be required for candidate gene by environment analysis [37]. The cumulative effect of childhood adversity was also a significant predictor of case/control status, consistent with previous studies that demonstrated multiple adversities were associated with increased risk for psychosis [3]. We have previously shown evidence of specificity between adverse childhood events and manifestations of psychotic disorders. Specifically, childhood sexual abuse, physical abuse and parental separation showed significant associations with positive psychotic symptom dimensions (e.g., delusions, hallucinatory behaviour) [38]. However, we found no difference in terms of age at onset, duration of untreated psychosis and overall clinical functioning between first-episode psychosis patients who reported childhood adversity and those who did not [39]. Due to limited sample size it was not possible in this study to conduct more fine-grained analyses of childhood adversity in interaction with PRS in relation to specific aspects of psychosis. Therefore, the pathoplastic influence of childhood adversity in conjunction with PRS on psychosis still needs further investigation.

Polygenic risk scores for schizophrenia did not increase exposure to, or reporting of, childhood adversity in cases and controls. This is in line with previous findings which showed no statistically significant correlations between PRS and childhood adverse events in both clinical and non-clinical samples [19,21]. Similar findings come from studies on candidate gene associations with history of childhood adversity in psychosis. For instance, Ajnakina et al. [12] found, in the same psychosis sample used for this study, that *FKBP5* genotype at rs1360780 locus was not associated with exposure to childhood adversity.

Moreover, our results show no moderation of the association between childhood adversity and psychosis by PRS for schizophrenia, using an additive model. These findings are in line with previous studies reporting that the effect of childhood trauma on later experience of psychotic symptoms was independent of proxy genetic liability to psychosis [5,9–11]. Overall, these results suggest that biological and environmental risk factors are both important in the etiology of psychosis but the effects of some forms of childhood adversity act largely independently of pre-existing genetic liability to increase risk of psychosis. This is in agreement with previous findings by Mullins et al. [20], which found no additive interaction between PRS and childhood adversity for recurrent depression. In contrast, Peyrot et al. [19] investigated whether the effect of polygenic risk scores on major depressive disorders was moderated by childhood trauma and found evidence for interaction as departure from both multiplicative and additive risks. Clearly, further studies are required to resolve these inconsistencies.

### Limitations

Despite this being a novel study, a number of limitations need to be taken into account. Firstly, the choice *a priori* of an additive model in our study was, along with Rothman et al [40], driven by a public health perspective (whether new cases of disease will be produced when individuals are exposed to two risk factors beyond what would be expected from the impact of the risk factors on their own). However, Zammit et al. [41] argued that multiplicative statistical models are likely to provide a better fit than additive ones for modelling the joint relationship of exposures on disease risk. Nonetheless, multiplicative models are considered more complex and error-prone in their estimation than additive models [42]. Given that these statistical models can give different results [42], our choice to utilise an additive model may have influenced our findings.

Secondly, as a retrospective study, our results are potentially sensitive to recall bias of childhood adversity. However, we used the CECA.Q [29] to improve the validity of the adverse experiences reported by participants. This questionnaire is designed to elicit concrete examples of adverse experiences and was read out loud to participants by trained researchers in order to improve the accuracy of the answers. Furthermore, we scored the severity of the responses in a standardised manner (see http://cecainterview.com/), using conservative cut-offs to ensure that only severe childhood adverse experiences were included in the analyses. Moreover, the use of retrospective assessment is common in studies investigating the role of childhood risk factors in clinically-relevant psychotic disorders, as it allows us to ask these important questions without reliance on excessively large (non-existent) samples followed up from childhood. Although some bias in retrospective reports has been demonstrated [43], it cannot be considered sufficient to render retrospective case–control studies of childhood experiences invalid [44]. Moreover, it has been shown that the impact of childhood adversity on psychosis is not confounded by the type of study design utilized [2] and psychosis patients are reliable and consistent over time in recalling histories of childhood adversity, regardless of the severity of current symptoms [31]. All of these factors increase the accuracy of an individual’s recall of past adverse experiences [44].

Although efforts were made to obtain a control sample that was representative of the local community population, it was not randomly selected and thus it is possible that this may have led to erroneous findings. The final sample of controls used in the current analyses was similar, according to the last UK census data, on a number of socio-demographic factors, such as gender and age, to the population that the cases came from (www.statistics.gov.uk/census/2001census). However, controls included in this study were more likely to be White British and with a higher level of education compared to cases, and we controlled for these demographic characteristics in all the analyses. In the current study, the rates of childhood adversity within the control sample were similar to those found in surveys of the UK general population [45], suggesting that this aspect of the control sample is unlikely to have affected the results.

Moreover, the sample was underpowered to detect the likely genetic and environmental interactions in psychosis. However, detection of ‘real’ interaction effects is dependent upon the accuracy with which the effects of each SNP included in the PRS are estimated within the ‘discovery sample’ and this is more likely when larger sample sizes are utilised [37]. As the schizophrenia PRS used in the current study was derived from the PGC [17] based on 34,241 schizophrenia cases and 45,604 controls, we can assume that this PRS was estimated with a reasonably high level of precision.

The participants in our subsample with PRS data were of white European descent, which may limit the generalizability of the present findings to different ancestral backgrounds. Principal components of ancestry were included as covariates in these analyses, as very subtle effects of population stratification at single SNPs could accumulate across the thousands of genetic variants in a polygenic score.

PRS capture a significant proportion of genetic liability as they are based on genome-wide SNP data [46]. However, PRS do not distinguish between SNPs that strengthen or reduce the association between childhood adversity and psychosis [19]. Jaffee and Price [47] have also warned that as polygenic risk scores aggregate information across thousands of SNPs they in essence provide a more “black box” genetic risk estimate than candidate genes. However, we have consistently shown in this sample that childhood adversity and familial liability did not combine synergistically to increase odds of psychosis beyond the effect of each individually [11].

Additionally, the cross-sectional design of this study limits causal inference. However, the absence of gene-environment correlations between PRS and childhood adversities in both cases and controls limits the possibility of bias from reciprocal causation.

Lastly, in this study we focused only on specific adverse childhood experiences measured by the CECA.Q. Other environmental risk factors, such as cannabis use [48] or trauma occurring in adulthood [49], which have previously shown strong associations with psychotic disorder, might act as confounders or moderators of the childhood adversity-psychosis association. Ideally, future studies should include larger samples and a range of environmental risk factors in order to further improve our understanding of the etiology of psychosis.

### Potential pathways from adversity to psychosis onset

The etiology of psychosis may be better understood by considering several layers of explanations, psychological as well neurobiological. Disturbances in childhood attachment, as a consequence of adverse childhood experiences, might lead to development of dysfunctional appraisals about the self and the world, such as hostile attributions of others' intentions, negative self-perceptions and lack of personal control over events, and these could be related to the onset and maintenance of psychotic phenomena [50]. Exposure to childhood adversity might also “sensitize” a person with genetic vulnerabilities to psychosis towards other stressors which, in turn, correspond to exaggerated emotional response at a behavioral level [51] and to an imbalance of the dopamine neurotransmission between prefrontal cortex and mesolimbic circuits [52], which is relevant to positive symptom formation [53]. In these ways, exposure to childhood adversity may be involved in the etiology of psychosis at a level that is distal to genetic vulnerability, which might be another reason why an interaction with PRS was not found in this study.

### Clinical implications

According to the liability threshold model, individuals within a population show a varying liability to disorder, and only those individuals whose liability exceeds the threshold will develop clinical illness [54]. In keeping with this model, the direct effects found within this sample of childhood adversity and a schizophrenia PRS on psychosis has several potential implications. The first is that the PRS for schizophrenia, accounting for around a quarter (23%) of trait variance [55], may help in predicting those at risk of psychosis, which potentially yields exciting opportunities for targeting specific clinical interventions, but also aids in planning possible future prevention programmes for individuals considered at risk and in improving public health strategies for psychosis [56]. Additionally, the high prevalence of childhood adversity amongst those experiencing psychosis, with the magnitude of such an association increasing in those reporting multiple adversities, emphasizes the need for early intervention programs to focus on these events, for example, by screening for childhood adversity, and offering specific treatments to reduce the high levels of emotional arousal and distress which result from the experience of early adversities. Therefore, it is imperative that clinicians enquire routinely about childhood adversities when they try to assist people experiencing clinically-relevant psychotic disorders.

Schizophrenia has been suggested to be a ‘pathway disease’ [57], and genetic evidence has shown glutamatergic, GABAergic, and dopaminergic signalling disruptions, in connection with immune and neurodevelopmental pathways. These pathways have been found to also be disrupted in victims of childhood adversity [58]. Future research should therefore be conducted using longitudinal studies with objective prospective environmental measures collected alongside genetic data [59] to better investigate the etiology of psychosis.

## Supporting Information

S1 Table Associations between the schizophrenia polygenic risk score and reports of childhood adversity adjusted only for principal components, gender and age at interview. (DOCX)

S2 Table Associations between the schizophrenia polygenic risk score and reports of childhood adversity in cases with schizophrenia-spectrum disorders. (DOCX)

S3 Table Interaction between the schizophrenia polygenic risk score and reports of childhood adversity on presence of psychotic disorders adjusted only for principal components, gender and age at interview. (DOCX)

S4 Table Interaction between the polygenic risk score and reports of childhood adversity on presence of schizophrenia-spectrum disorders. (DOCX)
