# Supplementary material for: Interplay between Schizophrenia Polygenic Risk Score and Childhood Adversity in First-Presentation Psychotic Disorder: A Pilot Study
Source: PLoS One. 2016 Sep 20;11(9):e0163319. doi: 10.1371/journal.pone.0163319 (PMC5029892; doi:10.1371/journal.pone.0163319)
Supplement: S1 Table — (DOCX) [file pone.0163319.s001.docx]

**S1 Table.** Associations between the schizophrenia polygenic risk score and reports of childhood adversity adjusted for principal components, gender and age at interview.

| **Gene–Environment correlation** | **Adjusted *b**** | **95% CI** | ***p*** |
| --- | --- | --- | --- |
| Psychosis Cases | 0.70 | -5.83-7.23 | 0.834 |
| Unaffected Controls | 1.07 | -3.29-5.43 | 0.631 |

**Notes:** CI, confidence interval. *b*, logistic regression coefficient.

*adjusted for ten principal components, gender and age at interview.
