# Supplementary material for: Interplay between Schizophrenia Polygenic Risk Score and Childhood Adversity in First-Presentation Psychotic Disorder: A Pilot Study
Source: PLoS One. 2016 Sep 20;11(9):e0163319. doi: 10.1371/journal.pone.0163319 (PMC5029892; doi:10.1371/journal.pone.0163319)
Supplement: S3 Table — (DOCX) [file pone.0163319.s003.docx]

**S3 Table.** Interaction between the schizophrenia polygenic risk score and reports of childhood adversity on presence of psychotic disorders adjusted for principal components, gender and age at interview.

| **Gene–Environment Interaction** | **Adjusted *b**** | **Std. Error** | ***p*** |
| --- | --- | --- | --- |
| PRS | **0.39** | 0.14 | **0.004** |
| Childhood adversity | 0.21 | 0.42 | 0.618 |
| PRS* Childhood adversity | 0.05 | 0.41 | 0.907 |

**Notes:** *b*, linear regression coefficient. Std. Error, Standard Error. Figures in bold indicate p<0.05.

*adjusted for ten principal components, gender and age at interview.
