# Supplementary material for: Interplay between Schizophrenia Polygenic Risk Score and Childhood Adversity in First-Presentation Psychotic Disorder: A Pilot Study
Source: PLoS One. 2016 Sep 20;11(9):e0163319. doi: 10.1371/journal.pone.0163319 (PMC5029892; doi:10.1371/journal.pone.0163319)
Supplement: S4 Table — (DOCX) [file pone.0163319.s004.docx]

**S4 Table.** Interaction between the polygenic risk score and reports of childhood adversity on presence of schizophrenia-spectrum disorders.

| **Gene–Environment Interaction** | **Adjusted *b**** | **Std. Error** | ***p*** |
| --- | --- | --- | --- |
| PRS | **2.85** | 0.99 | **0.005** |
| Childhood adversity | -0.58 | 3.71 | 0.876 |
| PRS* Childhood adversity | -1.33 | 3.76 | 0.724 |

**Notes:** PRS, Childhood adversity and their interaction were standardised by subtracting the mean and dividing by the standard deviation prior to fitting the model.

*b*, linear regression coefficient. PRS, polygenic risk score. Std. Error, Standard Error. Figures in bold indicate *p*<0.05.

*adjusted for two principal components (rather than 10 because of the small sample size), gender, age at interview and education level.
